# Experiences of nurses studying towards post-basic qualifications during COVID-19 in KwaZulu-Natal

**DOI:** 10.4102/hsag.v29i0.2609

**Published:** 2024-10-10

**Authors:** Dudu G. Sokhela, Kathleen Nokes, Penelope M. Orton

**Affiliations:** 1 Department of Nursing, Faculty of Health Sciences, Durban University of Technology, Durban, South Africa

**Keywords:** COVID-19 pandemic, online-based learning, post-basic nursing programmes, higher education, frontline nurses

## Abstract

**Background:**

The COVID-19 pandemic caused abrupt closure of schools including higher education institutions. The transition from in-person to virtual teaching platforms caused challenges for frontline nurses in post-basic programmes. Nurses were forced to divide their efforts between responding to the pandemic and their studies.

**Aim:**

The study aims to describe the experiences of nurses studying post-basic qualifications during COVID-19 in KwaZulu-Natal.

**Setting:**

The setting comprised virtual platforms such as closed social media groups (nursing colleagues and South African Society of Occupational Health Nurses Facebook) and Microsoft Teams or WhatsApp calls.

**Methods:**

A qualitative descriptive design using individual virtual interviews with eight nurses working in healthcare settings while studying post-basic qualifications. The framework was Fullan’s Change Theory in educational settings. Data were analysed using applied thematic analysis.

**Results:**

Four themes were identified; transitioning from in-person to online teaching and learning, challenges in clinical placements, support to achieve educational goals, and unique aspects of studying and caring for infected patients and 10 sub-themes emerged.

**Conclusion:**

Participants found benefits of online learning despite challenges with the Internet network. There was a shortage of mentors, and personal protective equipment in clinical settings; however, participants were cognisant of the duty to care for infected patients notwithstanding personal risk of infection.

**Contribution:**

Nurses were at the frontline of the pandemic yet facing challenges in clinical facilities and their studies. The study could contribute to understanding participants’ experiences, which might help with response planning in future pandemics.

## Introduction

The South African Nursing Council (SANC) regulates the education of nurses by prescribing the scope of practice which shapes nursing curricula and identifies competencies and functions that graduates are authorised to perform (SANC 2005). Nurses with basic training need to enter specialised nursing education programmes to gain the knowledge and skills needed for employment in specific fields such as primary care, occupational health, nursing management and intensive care. The South African Minister of Health announced the first confirmed COVID-19 positive case in March 2020 (NICD 2020) and the World Health Organization (WHO 2020) declared a global pandemic that same month. Lockdown regulations were imposed to curb the spread of infections and flatten the curve, including reducing mortality and morbidity as well as limiting the patient burden in the health system (Umviligihozo 2020). College campuses were abruptly closed across the globe (UNESCO 2020) which resulted in a tremendous impact on institutions of higher education (Williamson et al. 2021). Higher education providers rapidly transitioned from face-to-face to online teaching platform environments (Serhan 2020). Both students and the faculty were unprepared for the disruption of teaching and learning caused by the switch from face-to-face education to virtual blended learning platforms. The abrupt cancellation of in-person classes and the transition to online and virtual teaching platforms caused challenges for the frontline nurses (Ramos-Morcillo 2020). This was notably so for nurses who were studying in a post-basic programme which affected their ability to fully engage in their programme of study. This abrupt transition was particularly challenging in South Africa which experiences power cuts regularly, with 2022 recorded as the worst on record with 205 days of rolling blackouts (Ziady 2023) that blocked Internet access. Nursing students were forced to focus simultaneously on a healthcare environment where they were responding to the pandemic and their post-basic study.

## Background

Quintana and Quintana (2020) identify many pandemic-related factors that led to compounded student anxiety such as learning in their home environments and loss of support from face-to-face social interaction. However, many students appreciated the flexibility of online learning in terms of convenience in accessing lectures (Hasan & Khan 2020). Graduate students identify the importance of communication and the usefulness of different online platforms to communicate regularly with faculty, but missed the in-person interactions they had experienced before the pandemic (Nodine et al. 2022). Nurses attending post-graduate courses adapted to the transition from face-to-face to e-learning, although they initially had negative perceptions as it was not their preferred choice of learning method (Sarli et al. 2021). Frustration was generated when post-basic Clinical Nurse Specialist (CNS) students were uncertain whether they should continue to prepare for that role when they perceived that CNSs were being under-utilised in clinical settings (Ramelet 2022). Some students wanted to continue with their studies, although they were overwhelmed with demands to provide nursing care in the clinical areas and others were assigned to work in positions that were inconsistent with their curriculum (Prior et al. 2020). During this time, nurses had technical problems, while others expressed difficulty in paying attention during their lessons. These challenges were attributed to the lack of a conducive learning environment in a home environment, aggravated by the presence of their household members while studying (Hu et al. 2022).

In Africa in general, and South Africa in particular, the pandemic resulted in a leap into remote and blended learning, necessitated by the desire to complete the academic year and allow students to progress towards their graduation. The government of South Africa collaborated with several non-governmental entities to devise ways of continuing to deliver education. Several initiatives emerged based on online, mobile and social media platforms; but, despite these efforts, South Africa’s education system was ill-prepared (Dube 2020). University leaders across the continent and globally had to decide on various kinds of emergency measures and reimagine teaching and learning and student engagement and support to ‘save the academic year’ (Moja 2021). Prior research with South African nurses studying for specialist occupational health nursing qualifications had found significant computer anxiety, which was associated with less Internet self-efficacy, perceived ease of use and behavioural intention to use the computer for learning, pointing to potential challenges with the transition to online teaching platforms (Orton, Nokes & Hickey 2015). The study aimed to describe the experiences of nurses studying towards a post-basic qualification during the COVID-19 pandemic in KwaZulu-Natal.

## Research methods and design

### Study design

This study used a qualitative descriptive design to provide a rich description of participants about their experiences (Bradshaw, Atkinson & Doody 2017), using semi-structured individual virtual interviews to collect data. Data were analysed using applied thematic analysis as an inductive analysis of qualitative data that focuses on identifying and describing both implicit and explicit ideas within the data (Guest, Mac Queen & Namey 2012).

Fullan’s Change Theory in educational settings (2006) guided the study and emphasises the need to take action. There are seven core premises: (1) focus on motivation; (2) capacity building, with a focus on results; (3) learning in context; (4) changing context; (5) bias for reflective action; (6) tri-level engagement and (7) persistence and flexibility in staying the course. Because of the emergency features of the pandemic, the participants experienced an abrupt change in the learning context requiring them to rely on their motivation to continue their professional education and identify strategies to build capacity. A fuller appreciation of how frontline nurses continued their programmes of study will assist nurse educators in identifying barriers and facilitators presented by abrupt changes in the learning context.

### Research setting and participants

Participants were recruited through closed social media groups such as the Facebook pages for primary healthcare Nurses and Nursing colleagues, and South African Occupational Health Nurses (SASOHN). Nurses, working in healthcare settings while studying towards post-basic nursing qualifications during the pandemic, and who responded with interest to participate in the study, were conveniently sampled (Polit & Beck 2021) to participate in the study until data saturation was achieved. Only professional nurses who were working with infected patients during the COVID-19 pandemic while registered and studying towards a post-basic qualification were included.

### Data collection

Data were collected from January 2023 to March 2023. Eight participants responded to the recruitment post and signed a consent form via e-mail. Semi-structured interviews (Polit & Beck 2021) were conducted in English using Microsoft Teams and WhatsApp voice calls on the date and time agreed upon between the researcher and participant. Interviews, which lasted 40 min on average, were audio recorded with participants’ permission and transcribed verbatim by an independent transcriber after each interview.

### Data analysis

Applied thematic analysis was conducted to analyse data, by two qualitative analysis experts independently. Data were analysed concurrently with data collection. The verbatim transcriptions were read several times for researchers to be immersed in data for understanding and interpretation. Data were condensed into meaning units, which consisted of words, sentences and chunks of text, which contained aspects related to each other. These meaning units were reduced, and labelled with a code and entered into a codebook. Codes were sorted into categories and abstracted into themes. The two researchers met and agreed on the themes that emerged from the data. The independent coder confirmed themes and subthemes.

### Study rigour

Research rigour was achieved through trustworthiness to ensure the quality of the study:

- *Credibility*: This was ensured by using convenience sampling of participants who provided rich data because they had experienced studying during the pandemic. The same interview guide was used to collect data from all participants. The interviews were audio recorded and transcribed verbatim by an external transcriber.
- *Dependability*: An audit trail was developed by keeping audio-recorded data and written transcripts in a password-locked external hard drive which can be produced to verify data.
- *Confirmability*: Interpreted data were supported by direct quotes from raw data to eliminate subjectivity and bias of the research team. Raw data and the code book were further analysed independently by the third researcher.
- *Transferability*: The research report provides detailed and thick description such that other researchers can test its applicability to other contexts.

### Ethical considerations

The study was cleared by Institutional Research Ethics Committee of a university in Durban, KwaZulu-Natal (IREC 245/22). Participants voluntarily responded to social media recruitment agreeing to participate and providing signed informed consent through e-mail. During the interview, they were reminded about confidentiality and the right to withdraw at any time; codes were used instead of names on audio recordings, transcripts and reporting of findings. Audio-recorded data and written transcripts were kept in a password-locked external hard drive and will be kept for 5 years according to the university ethics rules.

## Results

Eight professional nurses, studying for post-basic qualifications during the pandemic who were also providing direct patient care, participated. The average participant was 36 years (range: 29–48 years); female; black African; worked an average of 10.5 years in nursing (range: 7–22 years). All the participants worked in primary healthcare settings, which, in the South African healthcare setting, is the first point of contact for entry into the healthcare system. Two of the participants were directly involved in screening and testing patients for COVID-19. The participants were experienced nurses, four of whom had post-basic qualifications and were furthering their education in other specialist nursing courses. Five were studying towards post-basic qualification in primary healthcare (clinical nurse practitioners), two were studying towards a nursing management qualification and one a Master’s in Public Health (a CNS in Occupational Health). Four of the participants described themselves as being in a nursing supervision or management role. Four themes emerged: transitioning from in-person to online teaching and learning; challenges applying specialisation content in clinical placements; support to achieve educational goals and unique aspects of studying and caring for patients during COVID-19 along with a number of subthemes.

### Theme 1: Transitioning from in-person to online teaching and learning

An online teaching and learning strategy was adopted by all educational institutions when they were forced to transition from face-to-face classroom teaching during the COVID-19 pandemic.

#### Subtheme 1.1: Internet network challenges

Poor network connectivity was a major challenge because of planned electricity blackouts or load shedding and inadequate data on their smartphones. The electric company scheduled blackouts at different times of the day in different geographical areas resulting in the students’ inability to log-in to the learning platform at designated times resulting in missed lectures. Participants said:

‘The main problem I had was network issue, it was very hard because the lecturer will give you the time Ok guys we gonna meet at eight o’clock on Teams … you will try to log in for about an hour, there’s no network.’ (P1, Female, 33 years old)

‘[*T*]he other barrier was network because you find that there’s no network when it’s time for class … when you try to find classes and you don’t have a network.’ (P6, Female, 39 years old)

These challenges resulted in participants not reaching their full potential and they did not perform as well as they would have if they were in a face-to-face classroom as evidenced by lower than typical course grades:

‘I was not scoring as high marks as I used to score.’ (P6, Female, 39 years old)

‘I’m a top-class student … ended up scoring little bit lower … wasn’t so happy about the results but I was glad that I made it.’ (P1, Female, 33 years old)

Electricity was crucial for Internet connectivity to enable participants to continue studying online. Load shedding resulted in inability of participants to log on to learning platforms.

#### Subtheme 1.2: Persistence and flexibility

Participants found that although they were distracted during lectures at home, study groups and the lecturers were a source of support and strength:

‘When you are online, there is the TV, there is the phone, you just get bored and you go to Facebook so it was difficult to adjust but then I remembered why I started doing this module because once you focus on achieving your goal then you have to remind yourself and say “I need to focus on this”.’ (P3, Female, 48 years old)

‘[*S*]ome of the learners who were familiar with the technology used to help me; I’ve asked my nephew because I not even aware that you can learn online.’ (P7, Female, 34 years old)

‘I had group of friends from the same class and I told them that “Eyi guys, it’s very hard. Please help me to figure out, how, to enter into Teams, how to go to the classroom, how to respond, how to check the slides that were uploaded”.’ (P1, Female, 33 years old)

Participants had challenges with attending class alone at home instead of a classroom setting, without the immediate support of colleagues and lecturers.

#### Subtheme 1.3: Finding benefits to online learning

Participants found that there were benefits to online learning such as flexibility. If students missed lectures or wanted to listen to it again, they used the recording which was particularly helpful for ill participants; they stated:

‘There was a particular time where I got really sick and I remember that we were writing our Pharmacology final exam and it was late for me to notify the faculty so I did struggle. I wrote the exam but unfortunately that was the day I got admitted to hospital and so the results came and I had failed.’ (P5, Male, 31 years old)

‘[*I*]n the middle of my course I fell pregnant it was challenging but at the same time I did benefit for doing the course online.’ (P7, Female, 34 years old)

For some participants, online learning provided relief and an escape from the reality of their challenging day-to- day work in clinical environments with infected patients:

‘I think classes were a good distraction from the outside work that we were doing you know. A day that you are off and you had to attend a class you enjoy, that day (sigh) that’s a relief.’ (P4, Female, 42 years old)

Attending classes offered a break from the realities of the COVID-19 pandemic and its effects on healthcare workers who were at the front lines providing nursing care during the pandemic.

### Theme 2: Challenges applying specialisation content in clinical placements

Nursing is a skill-based profession, and as such, participants had to complete a specific number of hours in a clinical setting under the supervision of a professional mentor.

#### Subtheme 2.1: Mentor shortages

Mentors were often not available because of being tested positive for COVID-19 and in isolation or too sick and admitted in hospital with COVID-19 infection:

‘It was very hard because the minute you pop in, you are expected to work like a normal employed person because they were short staffed … they ended up neglecting some of the students, there will be no one to supervise you. You’ll be expected to use your guidelines alone.’ (P1, Female, 33 years old)

‘Some of the staff got sick and they will be booked off, when it’s my practical day and I was very accepted, they were happy that at least there’s someone extra … they were benefitting from me because I’ve been in the clinic for few years.’ (P7, Female, 34 years old)

Nursing is skills based; hence, clinical placement required working under the mentorship of a qualified professional nurse. This was difficult during the pandemic.

#### Subtheme 2.2: Personal protective equipment challenges

Several participants found it hard to work wearing full personal protective equipment (PPE), as they felt like they could not breathe freely. In some facilities, only a few PPE items were available, and participants felt that they were inadequately protected. This affected participants’ learning, as they were preoccupied with thinking about contracting COVID-19 or taking the infection home to their families. Not only were there equipment shortages, the guidelines were adjusted depending upon available equipment as described by the participants:

‘They put a guideline on what to wear during COVID-19 pandemic, but when they run out of all of that they will then change the guideline and say “Now you can wear this”.’ (P4, Female, 42 years old)

‘Sometimes you feel that “Eyi now I can’t even breathe” but you had to be there because there‘s no one else.’ (P8, Male, 48 years old)

‘Patients were very heavy at that time more because normally we, nurse our patients who are mobile but at that time most of our patients were very sick, were immobile so we had to do almost everything for them. So, when you are doing everything for them and you are alone on this full PPE it used to be very hard.’ (P1, Female, 33 years old)

Protection was important for participants to prevent them from contracting infection. However, it created challenges and some felt that their learning was affected because they felt suffocated from the PPE.

### Theme 3: Getting support to achieve educational goals

During learning, students need support from their lecturers and fellow classmates to assist them to achieve their educational goals. This was more so during the pandemic when they were learning online.

#### Subtheme 3.1: Communicating with lecturers and classmates

Compared to face-to-face learning where discussions with lecturers was routine and answers immediate, participants reported delays in communication. The most commonly used strategies were e-mails (particularly with lecturers), WhatsApp and Microsoft Teams as that had been chosen as the learning platform. Participants said:

‘So, adaptation was not easy but we will make an appointment to teach us where we are lacking or where we not understanding they will always try to avail themselves to make the course to continue.’ (P5, Male, 31 years old)

‘Some lecturers record the lessons and share the recording afterwards otherwise we have to catch up from your colleagues, from the slide if you have any problems you can contact him or your colleagues. I think most of us were comfortable to contact each other versus the lecturer, you will send an email but it would take a while to come back and most people I think will attest to that as lecturers you don’t want to share your personal number with a student.’ (P2, Female, 33 years old)

‘We communicated via WhatsApp so we basically, would call to each other or since we were able to use the online platform of Teams, we would also use it if we want to study something or discuss something we will all go into the Teams as a group.’ (P5, Male, 31 years old)

The inability of participants to communicate face to face with lecturers posed a challenge which affected their learning.

#### Subtheme 3.2: Mentoring each other

Participants formed small groups where they met and discussed their studies and helped each other with schoolwork and technology. Participants shared:

‘Forming a group with my friends and made specific days that on this and that day go to university and use the library, so we book the rooms at library and then we meet there. And then in the library there was no problem with the network at all. Only the problem we had when there is load shedding but if there is no load shedding it was fine, the network was fine.’ (P1, Female, 33 years old)

‘Sometimes, we use library but it was very limited because of COVID, but we had a chance to use it.’ (P3, Female, 48 years old)

‘I know so many students who struggled a lot and since I was a class representative I had to phone other learners who were failing to accept calls, log in, who couldn’t submit whatever, who couldn’t see comments of assignments that have been marked which is basically a technical problem.’ (P4, Female, 42 years old)

Participants formed study groups with fellow students, which helped them to cope and gain hope to succeed.

### Theme 4: Unique aspects of studying and caring for patients during COVID-19

Participants had various experiences during the COVID-19 pandemic; they were nursing infected patients, which was scary on its own and had to study at the same time which affected their learning.

#### Subtheme 4.1: Multiple stressors

During the pandemic, participants were not spared from contracting the infection themselves. They were sick and had to be isolated while others were admitted to the hospital and some died. Participants worked long hours, had irregular schedules and were tired, particularly those who worked on night duty. Participants said:

‘You work hard you come back tired and you still have to go back the following day but that will then before you go to class you are going to prepare because somehow you need to do your own readings, you still have to submit assignments.’ (P8, Male, 48 years old)

‘One of my colleagues who was so sick she was in coma for like two weeks, she stayed in ICU for two weeks. I think we lost around six in our hospital.’ (P3, Female, 48 years old)

‘Most of them died then you will think that maybe you are next and you will think of your siblings and your kids and your family.’ (P7, Female, 34 years old)

Healthcare facilities were experiencing higher than normal patient volumes, and participants were expected to care for patients and also attend classes or be at clinical settings after working long hours.

#### Subtheme 4.2: Fear generated by uncertainty about COVID-19

While non-essential workers worked from home during various levels of lockdown, nurses could not avoid contact with infected people as they were classified as essential services. The participants expressed their fear of contracting the virus and dying from it:

‘I was scared to work with patients who were COVID positive, I would think about my baby my family, just wonder what’s going to happen if I die and leave my family.’ (P3, Female, 48 years old)

‘There were times where you really wanted to give up and say “I’m just stressing myself with this Masters will I ever qualify anyway? Will I be alive that time?” We were all very anxious and scared.’ (P2, Female, 33 years old)

‘In 2020 I remember when you go for tracing in other houses you will find that they are two people in that household who have died and there are few that are already sick, so that affected us emotionally like really and you become more scared.’ (P4, Female, 42 years old)

COVID-19 was a new infection and initially very little information was available which generated much anxiety among participants who had to nurse infected patients.

#### Subtheme 4.3: Impact on family members

Participants experienced different feelings regarding their families contracting infection from them. Participants said:

‘Sometimes you are busy studying and you find out that maybe your cousin has passed on because of the COVID, you will think that maybe you are the one who had a contact with the patient in the clinic and you went to see them and then they contacted COVID.’ (P7, Female, 34 years old)

This same participant felt scared for her young family and they sent the family away to minimise chances of infecting them. The participant stated:

‘It used to be so scary because you don’t know what is going to happen. I took my kids home – took them to Ulundi where it is my home because I was scared when I come back they will hug me, they want to spend the time with me. I was not willing to be with them for many hours because I was thinking that they might contract Covid from me.’ (P7, Female, 34 years old)

‘I tested positive for Covid but I never got sick. Together with my whole family we tested positive but nobody from us got sick and nobody died in my immediate family in my house nobody died and nobody got sick or admitted or anything like that.’ (P4, Female, 42 years old)

Participants had anxiety and felt guilty about infecting their families, more so as there was inadequate PPE to protect themselves.

## Discussion

The study describes the learning experiences of nurses who were providing healthcare and studying towards post-basic qualifications during the COVID-19 pandemic. The seven premises of Fullan’s Change Theory in educational settings (2006) addressed the four themes (Table 1).

**TABLE 1:** Themes, subthemes and application to Fullan’s Change Theory.

Themes	Subthemes	Fullan’s Change Theory (2006) seven premises
1. Transitioning from in-person to online teaching and learning	1.1. Challenges learning course content online 1.2. Internet network challenges 1.3. Persistence and flexibility 1.4. Finding benefits to online learning	Changing context (premise 4)
2. Challenges applying specialisation content in clinical placements	2.1. Mentor shortages 2.2. Personal protective equipment (PPE) challenges	Persistence and flexibility in staying the course (premise 7)
3. Getting support to achieve educational goals	3.1. Communicating with lecturers and classmates 3.2. Mentoring each other	Focus on motivation (premise 1) Capacity building, with a focus on results (premise 2) Bias for reflective action (premise 5)
4. Unique aspects of studying and caring for patients during COVID-19	4.1. Multiple stressors 4.2. Fear generated by uncertainty about COVID-19 4.3. Impact on family members	Learning in context (premise 3) Tri-level engagement (premise 6)

COVID-19, coronavirus disease 2019.

### Transitioning from in-person to online teaching and learning

Fullan’s 4th learning premise, changing context, applies in this theme. Prior to the pandemic, all learning occurred during in-person interactions with lecturers. While there was access to some learning platforms such as Blackboard, lecturers provided in-person classes which students preferred. The pandemic changed that context abruptly. Online learning was new to participants, and they reported having to adapt so that they could cope with this new way of learning. They found that being at home did not provide a conducive environment to study, as there were many distractions such as logging on social media, young babies demanding attention, house chores, and falling asleep. Faize and Nawaz (2020) also found that 12.7% students identified distractions during online sessions such as being disturbed by fellow students and family members most of whom were at home during lockdown. Although there were challenges and online learning was new to participants, they found some benefits such as saving on transport cost and travel time. As lectures were recorded, they could revisit the content at their own leisure for better understanding. Arifiati et al. (2020) also found that students were pleased that they could listen to class recordings at leisure in the comfort of their homes and saved on travel time and reduced travel expenses. The changing educational context engendered multiple challenges along with some benefits.

Nurses engage in continuing their formal professional nursing education by participating in professional development relevant to their areas of practice. There is often a need for healthcare professionals to renew and update their skills through continued professional development (Mlambo, Silen & McGrath 2021). Participants were not proficient with technology, had difficulty accessing and using it for schoolwork and, because of limited financial resources, did not have unlimited data. When this issue was recognised, some universities offered limited and unpredictable amounts of data to use for class work. Similar results were found in Ghana, where students were unfamiliar with Internet-based learning and had limited technology literacy (Owusu-Fordjour, Koomson & Hanson 2020). Besides challenges with technical skills, participants experienced poor connectivity related to the community where they lived and load shedding. This is a challenge in most countries where low bandwidth was reported, resulting in delays and poor connectivity during classes (Ferri, Grifoni & Guzzo 2020). Countries such as Indonesia reported unstable Internet networks and there was a lack of funds to purchase data resulting in obstacles to students’ online learning (Perwitasari, Astuti & Atmojo 2020).

### Challenges applying specialisation content in clinical placements

Fullan’s 7th learning premise, persistence and flexibility in staying the course, applies where participants demonstrated resilience which Fullan describes as persistence plus flexibility and stayed the course despite multiple challenges and resource scarcity. The second theme addressed challenges in clinical placements. To successfully complete the qualification, there were specific activities that students particularly needed to perform under the supervision of a qualified mentor at clinical facilities. There were several healthcare workers who were confirmed to be infected in South Africa and more than 50% of those were nurses (Grobler 2020) resulting in staff and mentor shortages. Without mentors, students were not able to complete the required clinical hours and the course in a timely fashion (McInnis, Schlemmer & Chapman 2021). The participants in this study were studying and employed full-time and faced PPE challenges especially shortages as, in some cases there were few items available, and participants could not don the complete set, and yet they could not abandon their duties. There was a reported global shortage of PPE, and this was stressful for both participants and mentors (Basen 2020). Other participants found it hard to work in full PPE, which was uncomfortable and difficult to breathe in. Shaukat, Ali and Razzak (2020) reported different skin eruptions, scarring and skin damage because of prolonged use of PPE.

### Getting support to achieve educational goals

Three of Fullan’s premises, focus on motivation (premise 1), capacity building with a focus on results (premise 2), and bias for reflective action (premise 5), support this theme. Fullan argues that all change requires motivation and participants were motivated to earn the nursing specialisation. They also needed to build their capacity in order to communicate through new methods using the Internet if they were going to achieve their goals. As people learn through continuous doing, reflection, inquiry and evidence, participants developed insights into successful methods of learning to achieve their goals. Communication with lecturers had changed during online learning and delays were stressful which was also found by Putri et al. (2020) and Purwanto et al. (2020). Participants preferred immediate responses as had been usual in face-to-face class. Challenges communicating with faculty were associated with students’ lower remote learning (Katz, Jordan & Ognyanova 2021). Communication among students was mainly through WhatsApp, which was easier, and Microsoft Teams which the university provided as a teaching platform. These platforms helped students to easily form groups where they could communicate and support one another in the absence of physical interaction and the shortage of mentors in clinical placement. Students learn better from others, who understand the challenges of being a student. Willing peer mentors in the third level of study were asked to support other students in lower levels (Rivenes Lafontan et al. 2023).

### The unique aspects of studying and caring for patients during COVID-19

Fullan’s third (learning in context) and sixth (tri-level engagement) learning premises apply in this theme. Learning in context actually changed the context, and participants reported increased coping strategies as they continued to face the day-to-day challenges. Tri-level engagement requires multi-level interventions which include the educational setting along with the local, provincial and country acceptance. Participants reported family, lecturer, university and community support which appreciated their unique stressors. The government made funds available to increase access to technology and lecturers used a variety of strategies to assist students to succeed. Participants were at the epicentre of the pandemic, taking care of infected patients who were commonly very sick and often highly infectious. They were anxious about contracting the virus particularly because of a lack of or poor protection and dying from the infection. There was little known about COVID-19 during the early days and there was a lot of fear and anxiety among nurses. Pandemics are known to cause fear and uncertainty among individuals (Khan & Huremović 2020). Participants faced a lot of challenges as they cared for infected patients and were perceived by communities as spreading the virus contracted from infected patients. They also feared taking the virus home to their families (Cai et al. 2020; Gılroy 2020). COVID-19 related stressors included economic stressors, effects on daily life and academic delays which were associated with increased anxiety levels of Chinese students during epidemics, while social support decreased anxiety (Huang, Xu & Liu 2020).

### Recommendations

Recommendation made by participants during interviews and recommendations from researchers are presented.

#### Recommendations from participants

Participants recommended what could be useful in future pandemics or similar crises, from their experience with the COVID-19 pandemic. Participants recommended the introduction of computer lessons as early as primary schools, which had not been the case with them. They had studied in educational programmes with no computer access in school especially in rural areas. Universities should provide laptops or tablets to students to enable them to access classes. Preparing systems for future pandemics and other similar crises is vital so that preparations do not only begin when the pandemic is upon us as it was the case with COVID-19.

#### Recommendations emerging from findings

It is recommended that online learning be adopted as a teaching method and blended into face-to-face lectures so that lecturers and students are prepared and ready to use it. Lessons learned from the COVID-19 pandemic should be used by different stakeholders including the Department of Health and Department of Education in readiness for future infectious emergencies such as readily accessible teaching and learning platforms and provision of adequate good quality PPE. Lecturers can share their contact information with students including cellphone numbers to keep open communication. Informal social networks could be established for supporting students such as WhatsApp. Flexibility includes conducting lectures on days and times that are suitable for students, not to stick to pre-approved lecture days and times during the pandemic or crisis. Clinical placement could be postponed during the height of the pandemic or crisis, and assessments conducted when students are more settled with online learning and work situation are better; this could help students to cope better. Lecturers could follow-up on students who were not completing assignments and offer them individual support and extend submission dates.

### Limitations

The study was qualitative and thus findings could not be generalised. The study included students who were studying towards a post-basic qualification; other nurses in training might have had similar or different experiences. In addition, the study was conducted in one province and reflected on the experiences of students from this province.

## Conclusion

During the COVID-19 pandemic, there were fears and anxiety about the future of nursing, and nurses surviving the infection. This future was unknown and participants in this study verbalised having these fears and almost giving up on their studies. Participants had doubts about their future health, including that of their families and relatives and their study plans, not knowing if they would survive the pandemic. They wanted to be available for those who were sick and needed to be nursed back to health. Participants seemed to gain some confidence as studies resumed and they formed groups among themselves, encouraged and supported each other to get through their studies. These challenges and anxieties were overshadowed by the greatest desire to continue with studying and completing the course, which was providing the foundation for specialisation practice.
